# A novel mathematical modeling with solution for movement of fluid through ciliary caused metachronal waves in a channel

**DOI:** 10.1038/s41598-021-00039-6

**Published:** 2021-10-18

**Authors:** Wasim Ullah Khan, Ali Imran, Muhammad Asif Zahoor Raja, Muhammad Shoaib, Saeed Ehsan Awan, Khadija Kausar, Yigang He

**Affiliations:** 1 School of Electrical Engineering and Automation, Wuhan University, Wuhan, 430072 China; 2 Department of Mathematics, COMSATS University Islamabad, Attock Campus, Kamra Road, Attock, Pakistan; 3 Future Technology Research center, National Yunlin University of Science and Technology, 123 University Road, Section 3, Douliou, Yunlin, 64002 Taiwan ROC; 4 Department of Electrical and Computer Engineering, COMSATS University Islamabad, Attock Campus, Kamra road, Attock, Pakistan

**Keywords:** Physics, Fluid dynamics, Mathematics and computing, Computational science

## Abstract

In the present research, a novel mathematical model for the motion of cilia using non-linear rheological fluid in a symmetric channel is developed. The strength of analytical perturbation technique is employed for the solution of proposed physical process using mectachoronal rhythm based on Cilia induced flow for pseudo plastic nano fluid model by considering the low Reynolds number and long wave length approximation phenomena. The role of ciliary motion for the fluid transport in various animals is explained. Analytical expressions are gathered for stream function, concentration, temperature profiles, axial velocity, and pressure gradient. Whereas, transverse velocity, pressure rise per wave length, and frictional force on the wall of the tubule are investigated with aid of numerical computations and their outcomes are demonstrated graphically. A comprehensive analysis for comparison of Perturb and numerical solution is done. This analysis validates the analytical solution.

## Introduction

**Figure 1:**
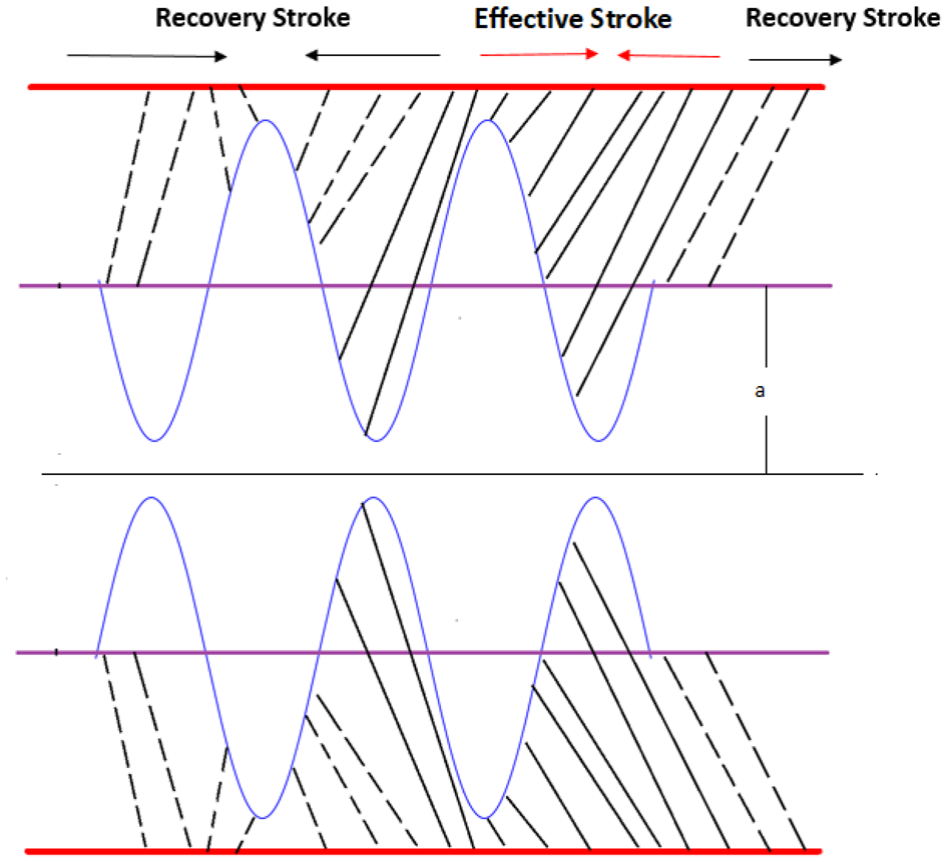
Metachronal wave pattern are exhibited due to ciliary wave motion.

Three types of cells movement in human beings and in various animals have been observed, namely: (i) Amoeboid movement (ii) Muscular movement (iii) Ciliary movement. (i) Amoeboid movement: Movement by pseudopodia (pseudo means false and podia means feet). Cells in human body which exhibit amoeboid movement are: Leucocytes (White blood cells), Macrophages (immune system). (ii) Muscular movement: In the human body, this movement is shown by movement of limbs and movement of jaws, tongue, eyelids etc. (iii) Ciliary movement: Movement by numerous hair-like structure. The regions in human body which exhibit this type of movement are: Respiratory tract lined by ciliated epithelium and reproductive system for the movement of fluid. In respiratory tract, cilia are present in trachea which helps to inhale oxygen inside and stop dust and other harmful particles and remove them outside. Cilium is a short microscopic hairlike vibrating structure, found in large numbers on the surface of certain cells, or in some protozoans and other small organisms, providing propulsion. Cilia consist of plasma membrane, peripheral microtubules, central microtubule, radial spoke, and liner and they contain basal body base. They are found in almost all animals, and they provide locomotion to moving fluid along internal epithelial tissue and ciliated protozoans. In some animals, many cilia may fuse together to form cirri. “The cirri are stiff structures and are used as something like legs”. According to Lardner and Shack^1^, movement of cilia plays an important role in many physical procedures i.e., reproduction, rotation, inhalation, alimentation and locomotion. The rehological fluid motion due to ciliary caused metachoronal wave is exhibited in Fig. 1.

The physiological aspects of ciliary transport has been studied by Lodish et al.^2^. Akbar et al.^3^ presented a non-Newtonian physiological fluid motion in a channel consisting of two parallel oscillating walls. Sadaf and Nadeem^4^ investigated fluid motion produced by cilia and pressure gradient through a curved channel along with heat transfer and radial magnetic field effects. Akram et al.^5^ examined the combined effects of peristalsis along with electroosmosis induce flow of silver-water nanofluid and silicon dioxide–water nanofluid for a permeable channel. Riaz et al.^6^ carried out a computational investigation which is applied on the peristaltic propulsion of nanofluid flow for a permeable rectangular duct. The impact of Hall effect on the peristaltic motion of Johnson-Segalman fluid in a heated channel with elastic boundaries has been investigated by Javed^7^. Bhatti et al.^8^ focused on transport phenomena of particle-fluid motion through an annular gape region. There are two groups in which cilia are divided, namely motile and non-motile cilia. Non-motile are identified as primary cilia. Single non motile cilium is found in nearly all cells, and plays a role in sensory functions. We will discuss importance of motile cilia here, which don’t beat casually(randomly), but through a synchronized way. The behaviour of cilia holds some vital features of ciliated epithelium. Mucus layer is present on the top of motile cilia. Motile cilia are rarely exists, and they are found in respiratory and reproductive system as well as in brain and spinal cord. Rivera^9^ narrated several explanations and implications about cilia gill (respiratory organ) for aquatic species, which may be enlisted as follow: (i) The beating rate of all the cilia is fairly unvarying, in any given tissue. (ii) The flagellation of cilium and cilium on the adjusted cells stay greatly synchronized. (iii) Certain movements from one to another place are created, whereas a movement in a given row of cells can be defined as a movement involving a beat arrangement, from one line to another line of cilia and so on.

As it well addressed metachronal rhythm provides concise flow of water with time through the surface of cilia, or probable it is unrealistic to arise synchronous beat over large area, it’s thought that cilia do not beat in a synchronous way, but in a systematic way. Nevertheless, along the surface of cilia metachronal rhythm may vary their shape, and this variation depending on whether the metachronal rhythm is accelerated toward the operative lash of the ciliary beat, or cilia beat is perpendicular to the lash of wave movement or may pass in the reverse direction of the of actual lash of beat and then in opposite way of flow. Very limited data is available about metachronal rhythm velocities, frequencies and wave-lengths. A 2-dimensional viscous fluid transport of nanoparticles past a channel along ciliated walls is investigated by Nadeem and Hina^10^. According to previous observations which revealed through experiments, many biological fluids exhibit non-Newtonian behavior^11–21^. For the simple Newtonian fluid non-satisfactory outcomes are analyzed. For rheological fluid transport, some of the researchers used Power-Law Model^14–17,22^. This model mostly rely on the fluid behavior index *n* due to its rheological nature.

Lauga and Powers^23^, Cordero and Lauga^24^ emphasized on biophysical and mechanical aspect for locomotion of microorganisms. They mathematically explored the importance of shear-dependent viscosities for the locomotion of flagella and cilia induced motion because of metachronal rhythm. Ciliary motion has been studied by the researchers by utilizing two models i.e. (i) Envelop model (ii) Sublayer model. The envelope model approach has edge over the other model because of metachronal beats on cilia layer by overlooking the particulars of sub-layer dynamic forces. Furthermore, the envelope model can be used for quantitative analysis e.g., for comparing swimming velocities that are mathematically gauged with available data, which are recorded in water for numerous microorganisms^24^. In addition, the perturbation method can be used for analysis and systematic study of non-Newtonian effects. Recently, the power-law fluid because of ciliary motion has been studied by Siddique et al.^25^, in the unlimited channel. They showed that power-law fluid provides outcomes, which are nearer to estimated value $6\times 10^{-3}$ ml/h. One can find some of recent work^27–30^.

A problem (non-linear) of Pseudo plastic fluid transference produced through cilia beating sequence of cilia in a given row of cells from row-to-row and metachronal wave movement is of great importance. On basis of mathematical study, rate of flow, velocity and pressure change will be calculated. Han et al.^31^ demonstrated that ultracold atomic may initiate a super solid phase while interacted with spin-orbit and a spin-dependent array of potential. Li et al.^32^ studied multi variant solutions for the polar and ferromagnetic of the universal model of a spinor model of the Bose-Einstein condensate. Wen et al.^33^ emphasized on matter rogue wave in Bose-Einstein condensates with dynamic inter-atomic contact with the aid of approximate and computation techniques. Transport of Non-Newtonian fluid in the ductus efferentes has been extensively studied by^34–36^.

There are some useful applications of artificial cilia in microfluidics (a) closed-loop channel and (b) open-loop channel with artificial magnetic cilia used in microfluidic pumping and also for flow control in tiny bio-sensors^26^. Cilia in the following study will not be assumed as flagella but ciliated epithelium. The key aspects of current investigation may me expressed in term salient features as:

- To emphasis on the motion of cilia induce mechanism using non-linear rheological fluidic system past a symmetric channel.
- Viewing the physiology of the problem, a mathematical model is developed using low Reynolds number and long wave length approximation.
- Analytical expressions for stream function, axial velocity, concentration and temperature profiles and pressure gradient are explored, whereas an attempt is made to numerically compute transverse velocity, pressure rise and friction force.
- Physical impact of crucial flow parameters are examined on the stream function, velocity profile, concentration, temperature profile, pressure gradient, pressure rise, frictional force on the walls the channel.

Further the paper is designed in following systematic manner.

## Modeling of the rheological problem

A two dimensional, incompressible, rheological Pseudo plastic nanofluid in a symmetric channel is analyzed. A cilia induced flow in a channel having infinite length is considered. The inside walls of the system based channel are assumed to be populated with a ciliated carpet. Further, it is assumed that flow is initiated by systematic beating of cilia which creates a metachronal wave, at the right side of the channel. We can identify a reference frame (${\overline{X}}$, ${\overline{Y}}$), in a manner that ${\overline{X}}$-axis lies along the center of the channel and ${\overline{Y}}$-axis is in the transverse direction. Both the plates are at 2 h apart.

For physiological problem we take velocity profile in the following form

1 $$\begin{aligned} \mathbf {V}=\left[ {\overline{U}}\left( {\overline{X}},{\overline{Y}},{\overline{t}} \right) ,{\overline{V}}\left( {\overline{X}},{\overline{Y}},{\overline{t}}\right) ,0 \right] , \end{aligned}$$

where ${\overline{U}}$ and ${\overline{V}}$ components of fluidic velocity profile in the axial and transverse direction, respectively.

Considering ${\overline{S}}$ the stress tensor for pseudo plastic fluid model is given,

2 $$\begin{aligned}&\overline{\mathbf{S }} + {\overline{\lambda }}_{1}. \overline{\mathbf{S }}^{\triangledown }+\dfrac{1}{2}({\overline{\lambda }}_{1}-{\overline{\mu }}_{1})(\overline{\mathbf{A }}_{1}\overline{\mathbf{S }}+\overline{\mathbf{S }} ~\overline{\mathbf{A }}_{1})=\mu \overline{\mathbf{A }}_{1} \end{aligned}$$

3 $$\begin{aligned}&\overline{\mathbf{S }}^{\triangledown }=\dfrac{d\overline{\mathbf{S }}}{dt}-\overline{\mathbf{S }}~\overline{\mathbf{L }}^{T}-\overline{\mathbf{L }}~{\overline{S}} \end{aligned}$$

4 $$\begin{aligned}&\overline{\mathbf{A }}_{1}=\overline{\mathbf{L }}+\overline{\mathbf{L }}^{*}, \end{aligned}$$

where

5 $$\begin{aligned} \overline{\mathbf{L }}=\nabla \overline{\mathbf{V }}, \end{aligned}$$

where $\mu$ represents viscosity of the fluid, $\overline{\mathbf{S }}^{\triangledown }$ is upper-convected derivative, $\mathbf{A }_{1}$ is used for Rivlin-Ericksen tensor of first type, $\dfrac{d}{dt}$ is material derivative and ${\overline{\mu }}_{1}$ and ${\overline{\lambda }}_{1}$ are the relaxation times. Continuity, momentum, energy and concentration equations may narrated in the vector form as:

6 $$\begin{aligned}&\nabla .\overline{\mathbf{V }}=0, \end{aligned}$$

7 $$\begin{aligned}&\rho _{f}\frac{d{\overline{\mathbf{V }}}}{dt}=-\nabla {\overline{P}}+\nabla {\overline{\mathbf{S }}}, \end{aligned}$$

8 $$\begin{aligned}&\frac{d{\overline{\mathbf{T }}}}{dt}=\overline{\mathbf{S }}.\overline{\mathbf{L }}+\alpha _{1}\nabla ^{2}\overline{\mathbf{T }}+\tau \left[ D_{B}\nabla \overline{\mathbf{C }}.\nabla \overline{\mathbf{T }}+\dfrac{D_{T}}{T_{m}}\nabla \overline{\mathbf{T }}.\nabla \overline{\mathbf{T }}\right] \end{aligned}$$

9 $$\begin{aligned}&\frac{d{\overline{\mathbf{C }}}}{dt}=\dfrac{D_{T}}{T_{m}}\nabla ^{2}\overline{\mathbf{T }}+D_{B}\nabla ^{2}\overline{\mathbf{C }} \end{aligned}$$

where $\rho _{f}$ is the density of fluid, ${\overline{P}}$ is the pressure, $\tau$ is the ratio of heat capacity of nano particle material to fluid, ${\overline{S}}$ is the extra stress tensor, $D_{B}$ is the Brownian diffusion coefficient, $D_{T}$ is the thermophoretic diffusion coefficient, $T_{m}$ is the fluid mean temperature and $\alpha _{1}$ is the thermal diffusivity.

In order to investigate the problem in a better and simple method, laboratory frame is shifted to wave frame, the transformations from moving frame of wave frame are $(\bar{X},\bar{Y}),$

$$\begin{aligned} \bar{u}(\bar{x},\bar{y}) & = \bar{U} - c,\bar{v}(\bar{x},\bar{y}) = \bar{V},\;\;\;\bar{x} = \bar{X} - c\bar{t},\bar{y} = \bar{Y},\bar{p}(\bar{x},\bar{y}) = \bar{P}(\bar{X},\bar{Y},\bar{t}). \\ \lambda _{1} & = \frac{{\bar{\lambda }_{1} c}}{{d_{1} }},x = \frac{{\bar{x}}}{\lambda },y = \frac{{\bar{y}}}{a},\;\;\;\delta = \frac{a}{\lambda },h = \frac{H}{a},t = \frac{{c\bar{t}}}{\lambda },v = \frac{{\bar{v}}}{c},\;\;\;Re = \frac{{\rho ca}}{\mu },\sigma = \frac{{\bar{C} - C_{0} }}{{\bar{C}_{1} - \bar{C}_{0} }},\;\;\;\Pr = \frac{\nu }{\alpha },u = \frac{{\bar{u}}}{c},\theta = \frac{{\bar{T} - T_{0} }}{{T_{1} - T_{0} }},N_{t} = \frac{{\tau D_{T} (T_{1} - T_{0} )}}{{\alpha T_{m} }},\;\;\;N_{b} = \frac{{\tau D_{B} (C_{1} - C_{0} )}}{\alpha },\;\;\;S_{{ij}} = \frac{{a\bar{S}_{{ij}} }}{{\mu c}},\;\;\;\mu _{1} = \frac{{\bar{\mu }_{1} c}}{a},u = \frac{{\partial \psi }}{{\partial y}},v = - \delta \frac{{\partial \psi }}{{\partial x}}, \\ \end{aligned}$$

where *Re* is the Reynold number, $N_{t}$ is thermophoresis number, $N_{b}$ is Brownian motion and *Pr* is the Prandtl number.

In moving wave frame, by capitalizing the non-dimensional parameter along with low Reynolds number and long wavelength approximation the equations of motion^38,39^, take the form as:

10 $$\begin{aligned}&\frac{\partial ^{4}\psi }{\partial y^{4}}-\zeta \frac{\partial ^{2}}{\partial y^{2}}\left( \frac{\partial ^{2}\psi }{\partial y^{2}}\right) ^{3}=0, \end{aligned}$$

11 $$\begin{aligned}&\frac{\partial p}{\partial x}=\frac{\partial }{\partial y}\left[ \psi _{yy}\left\{ 1-\zeta \left( \frac{\partial ^{2}\psi }{\partial y^{2}}\right) ^{2}\right\} \right] , \end{aligned}$$

12 $$\begin{aligned}&\frac{\partial ^{2}\theta }{\partial y^{2}}+PrN_{b}\frac{\partial \theta }{\partial y}\frac{\partial \sigma }{\partial y}+PrN_{t}\left( \frac{\partial \theta }{\partial y}\right) ^{2}+PrEc\left( \frac{\partial ^{2}\psi }{\partial y^{2}}\right) ^{2}=0, \end{aligned}$$

13 $$\begin{aligned}&\frac{N_{t}}{N_{b}}\frac{\partial ^{2}\theta }{\partial y^{2}}+\frac{\partial ^{2}\sigma }{\partial y^{2}}=0, \end{aligned}$$

with the relaxation time $(\lambda _{1}^{2}- \mu _{1}^{2})= \zeta$ and the corresponding dimensionless boundary conditions^40,41^ for cilia induced rheological fluid model are

14 $$\begin{aligned}\frac{\partial ^{2}\psi }{\partial {y ^{2}}}=0,\psi =0,\theta =0,\sigma =0, \end{aligned}$$

at $$y=0$$

15 $$\begin{aligned} \frac{\partial \psi }{\partial y}=u_{h}=-1-2\alpha \delta \epsilon cos(2\pi x), \psi =\frac{F}{2},\theta =1,\sigma =1, \end{aligned}$$

at $$h=1+ cos(2\pi x)$$.

## Solution methodology

It is hard to get the exact solution of the Eqs. (10–13), so we will employ perturbation method for small parameter $\zeta$

16 $$\begin{aligned} \psi= & {} \psi _0+\zeta \psi _1+\zeta ^{2}\psi _2^{2}+... \end{aligned}$$

17 $$\begin{aligned} \sigma= & {} \sigma _0+\zeta \sigma _1+\zeta ^{2}\sigma _2^{2}+... \end{aligned}$$

18 $$\begin{aligned} \theta= & {} \theta _0+\zeta \theta _1+\zeta ^{2}\theta _2^{2}+... \end{aligned}$$

Expressions using perturb solution up to second order for stream function, concentration, and temperature are

19 $$\begin{aligned} \psi&=\frac{3~Fh^{2}~y-2h^{3}~u_{h}~y-F~y^{3}+2h~u_{h}~y^{3}}{4h^{3}} +\zeta \frac{27(-F+h~u_{h})^{3}~y~(h^{2}-y^{2})^{2}}{20~h^{9}} \nonumber \\&\quad +\zeta ^{2} \frac{1}{385000~h^{27}}~19683~(-F+h~u_{h})^{9}~y~(h^{2}-y^{2})^{2}(767~h^{6}+1150~h^{4}~y^{2}-2625~h^{2}~y^{4}+3500~y^{6}), \end{aligned}$$

20 $$\begin{aligned} \sigma&={\gamma _{1}}-\frac{{\gamma _{4}N_{t}}}{{ N_{b}}}+{\gamma _{2}}y+\frac{{N_{t}}\left( 4{\gamma _{2} }^{2}{\gamma _{3}}e^{-{\gamma _{2}N_{b}}\Pr y}{N_{b}} ^{2}\Pr ^{2}+\frac{3{Ec}(F-2h{u_{h}})^{2}y(6+{\gamma _{2}N_{b}}\Pr y(-3+{\gamma _{2}N_{b}}\Pr y))}{h^{6}}\right) }{4{ \gamma _{2}}^{3}{N_{b}}^{4}\Pr ^{3}}+\zeta \Bigg( {\delta _{1}}+{\delta _{2}}y-\nonumber \\&\quad \frac{1}{175{ \delta _{2}}^{7}h^{18}{N_{b}}^{8}\Pr ^{7}}{N_{t}}\left( -175 {\delta _{2}}^{6}{\delta _{3}}e^{-{\delta _{2}N_{b}}\Pr y}h^{18}{N_{b}}^{6}\Pr ^{6}+175{\delta _{2}}^{7}{\delta _{4}}h^{18}{N_{b}}^{7}\Pr ^{7}\right. \nonumber \\&\quad -91854{Ec}\left( 1000-40{\delta _{2}}^{2}h^{2}{N_{b}} ^{2}\Pr ^{2}+{\delta _{2}}^{4}h^{4}{N_{b}}^{4}\Pr ^{4}\right) (F-h{u_{h}})^{6}y+45927{\delta _{2}EcN_{b}}\Pr \nonumber \\&\quad \left( 1000-40{\delta _{2}}^{2}h^{2}{N_{b}}^{2}\Pr ^{2}+{ \delta _{2}}^{4}h^{4}{N_{b}}^{4}\Pr ^{4}\right) (F-h{u_{h}} )^{6}y^{2}-15309{\delta _{2}}^{2}{EcN_{b}}^{2}\Pr ^{2} \nonumber \\&\quad \left( 1000-40{\delta _{2}}^{2}h^{2}{N_{b}}^{2}\Pr ^{2}+{ \delta _{2}}^{4}h^{4}{N_{b}}^{4}\Pr ^{4}\right) (F-h{u_{h}} )^{6}y^{3}-153090{\delta _{2}}^{3}{EcN_{b}}^{3}\Pr ^{3} \nonumber \\&\quad \left( -25+{\delta _{2}}^{2}h^{2}{N_{b}}^{2}\Pr ^{2}\right) (F-h{u_{h}})^{6}y^{4}+30618{\delta _{2}}^{4}{EcN_{b}} ^{4}\Pr ^{4}\left( -25+{\delta _{2}}^{2}h^{2}{N_{b}} ^{2}\Pr ^{2}\right) \nonumber \\&\quad \left. (F-h{u_{h}})^{6}y^{5}+127575{\delta _{2}}^{5}{ EcN_{b}}^{5}\Pr ^{5}(F-h{u_{h}})^{6}y^{6}-18225{\delta _{2}} ^{6}{EcN_{b}}^{6}\Pr ^{6}(F-h{u_{h}})^{6}y^{7}\right) \Bigg) \end{aligned}$$

21 $$\begin{aligned} \theta&={\gamma _{4}}-\frac{4{\gamma _{2}}^{2}{ \gamma _{3}}e^{-{\gamma _{2}N_{b}}\Pr y}{N_{b}}^{2}\Pr ^{2}+ \frac{3{Ec}(F-2h{u_{h}})^{2}y\left( 6-3{\gamma _{2}N_{b} }\Pr y+{\gamma _{2}}^{2}{N_{b}}^{2}\Pr ^{2}y^{2}\right) }{h^{6}} }{4{\gamma _{2}}^{3}{N_{b}}^{3}\Pr ^{3}}\nonumber \\&\quad \zeta \Bigg(\frac{1}{175{\delta _{2}}^{7}h^{18}{N_{b}} ^{7}\Pr ^{7}}\left( -175{\delta _{2}}^{6}{\delta _{3}}e^{-{ \delta _{2}N_{b}}\Pr y}h^{18}{N_{b}}^{6}\Pr ^{6}+175{\delta _{2}}^{7}{\delta _{4}}h^{18}{N_{b}}^{7}\Pr ^{7}-91854{Ec }\right. \nonumber \\&\quad \left( 1000-40{\delta _{2}}^{2}h^{2}{N_{b}}^{2}\Pr ^{2}+{ \delta _{2}}^{4}h^{4}{N_{b}}^{4}\Pr ^{4}\right) (F-h{u_{h}} )^{6}y+45927{\delta _{2}EcN_{b}}\Pr \nonumber \\&\quad \left( 1000-40{\delta _{2}}^{2}h^{2}{N_{b}}^{2}\Pr ^{2}+{ \delta _{2}}^{4}h^{4}{N_{b}}^{4}\Pr ^{4}\right) (F-h{u_{h}} )^{6}y^{2}-15309{\delta _{2}}^{2}{EcN_{b}}^{2}\Pr ^{2} \nonumber \\&\quad \left( 1000-40{\delta _{2}}^{2}h^{2}{N_{b}}^{2}\Pr ^{2}+{ \delta _{2}}^{4}h^{4}{N_{b}}^{4}\Pr ^{4}\right) (F-h{u_{h}} )^{6}y^{3}-153090{\delta _{2}}^{3}{EcN_{b}}^{3}\Pr ^{3} \nonumber \\&\quad \left( -25+{\delta _{2}}^{2}h^{2}{N_{b}}^{2}\Pr ^{2}\right) (F-h{u_{h}})^{6}y^{4}+30618{\delta _{2}}^{4}{EcN_{b}} ^{4}\Pr ^{4}\left( -25+{\delta _{2}}^{2}h^{2}{N_{b}} ^{2}\Pr ^{2}\right) \nonumber \\&\quad \left. (F-h{u_{h}})^{6}y^{5}+127575{\delta _{2}}^{5}{ EcN_{b}}^{5}\Pr ^{5}(F-h{u_{h}})^{6}y^{6}-18225{\delta _{2}} ^{6}{EcN_{b}}^{6}\Pr ^{6}(F-h{u_{h}})^{6}y^{7}\right) \Bigg) \end{aligned}$$

In Eqs. (20 and 21) $\gamma _{1},...,\gamma _{4}$ and $\delta _{2},...,\delta _{4}$ are variable expressions, and their values are incorporated in the “Appendix”.

## Velocity profile

Using the relation $u=\frac{\partial \psi }{\partial y}$ one may obtain expression for axial component of velocity from Eq. (19) as:

22 $$\begin{aligned} u&=-\frac{{u_{h}}}{2}+\frac{3\left( 2h{u_{h}} y^{2}+F(h-y)(h+y)\right) }{4h^{3}}+\frac{27(-F+h{u_{h}})^{3}\left( h^{4}-6h^{2}y^{2}+5y^{4}\right) \zeta }{20h^{9}}\nonumber \\&\quad +\frac{19683(-F+h{u_{h}})^{9}(h-y)(h+y)\left( 767h^{8}-385h^{6}y^{2}-21175h^{4}y^{4}+48125h^{2}y^{6}-38500y^{8}\right) \zeta ^{2}}{385000h^{27}} \end{aligned}$$

Pressure gradient is gathered as

23 $$\begin{aligned} \frac{dp}{dx}=-\frac{3({F_{0}}-2h{u_{h}})}{2h^{3}}-\zeta \left( \left. \frac{3\left( -27{F_{0}}^{3}+20F_{1}h^{4}+162{ F_{0}}^{2}h{u_{h}}-324{F_{0}}h^{2}{u_{h}}^{2}+216h^{3} {u_{h}}^{3}\right) }{40h^{7}}\right) \right. \end{aligned}$$

One may obtain the expression by integration the continuity equation

24 $$\begin{aligned} v=-\int \frac{\partial u}{\partial x}dy+c, \end{aligned}$$

The pressure rise per Wavelength is explored as

25 $$\begin{aligned} \Delta p_{\lambda }=\int _{0}^{1}\frac{dp}{dx}dx. \end{aligned}$$

$F_{\lambda }$ is the frictional force which can be obtained as

26 $$\begin{aligned} F_{\lambda }=\int _{0}^{1}h\left( -\frac{dp}{dx}\right) dx. \end{aligned}$$

It is hard to get the analytical expression for velocity component in the transverse direction, pressure rise, and frictional force. So, they are computed numerically and their results are exhibited graphically.

## Analysis of the physical problem

Cilia has numerous applications, it has been investigated by various researchers that cilia are responsible for fluid locomotion in ductus efferentes. Ductus efferentes are various small tubes which establishes important relation between testis and epididymis. Composition of these tubes is that these tubes are consists of single layer epithelium, this structure is strengthened by layer of uniform muscle and adjoining tissue^1–17^. These tubes transport sperm via rate testis to epididymis and recollect large quantity of fluid arising from rete testis. Ductus efferentes epithelium consists of both ciliated non ciliated cells. Besides this ciliary activity has great significance in the transport of protozoa in which locomotion is done via cilia. Outcomes of current investigation may be significant cilia dependent actuator in the function of biosensors and in drug delivery systems. It is important to mention here that not too much information is available about rate to due ciliary caused flows^1–37^. For the purpose of quantitative investigation, we provide estimate of different physical quantities related to physical study of fluid rheology of cilia induce flows. We have used following data to study rheological fluid motion. $\varepsilon =0.1$ to 0.2, $\alpha =0.2$ to 1, $\delta =0.1$ to 0.1, $Q=0.1$ to 0.5.

**Figure 2:**
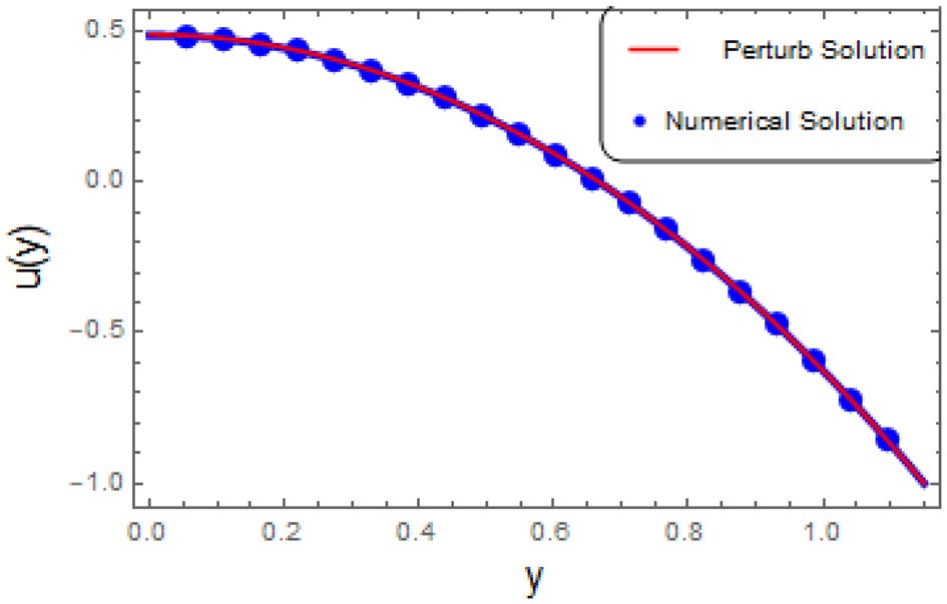
Plot of Perturb with numerical solution with $\zeta =0.01,\alpha = 0.2$, $\varepsilon = 0.15$, $\delta =0.01$, $F= 0$, $x= 1$.

**Figure 3:**
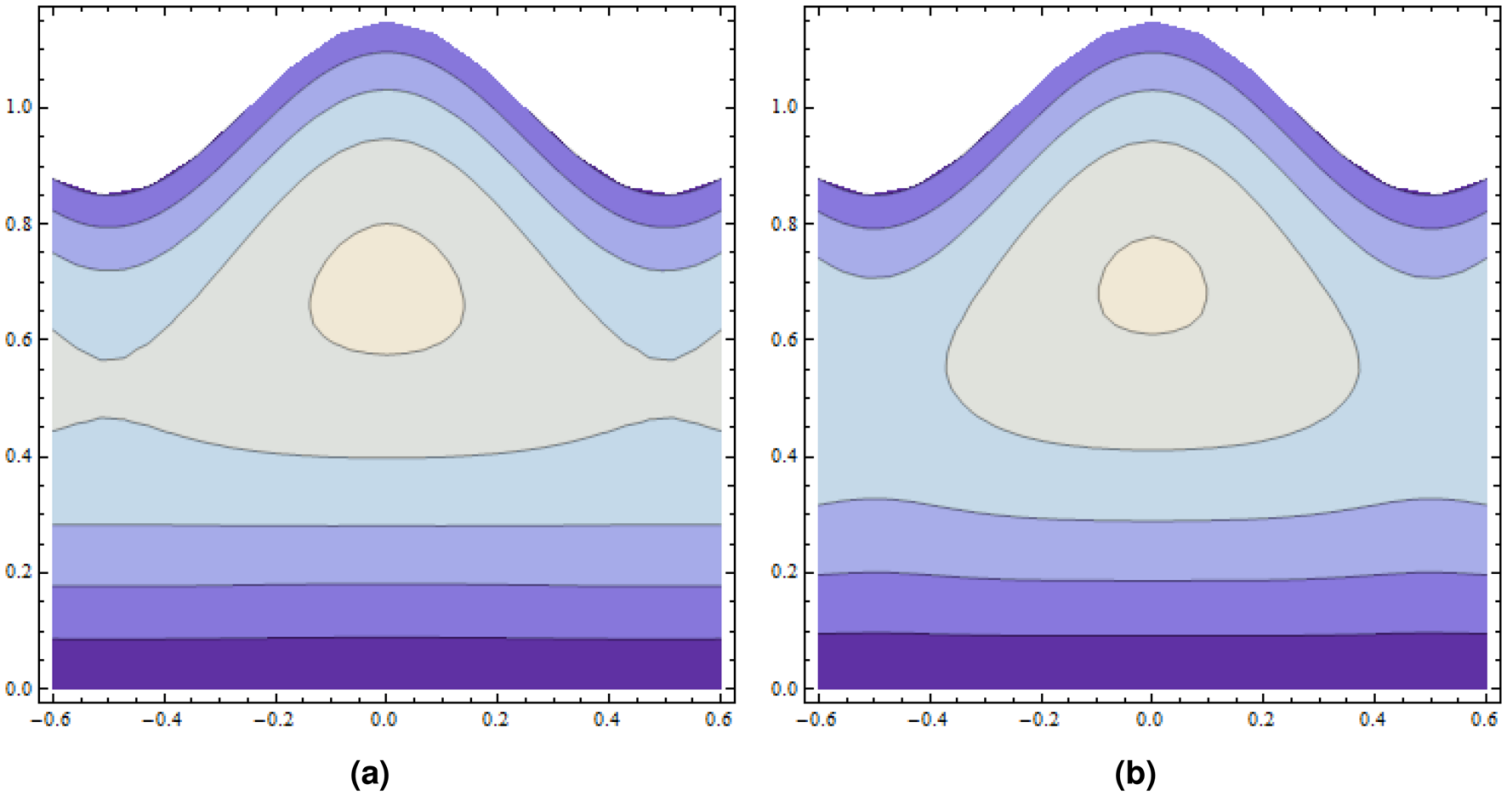
Plots of several stream lines with $\alpha =0.2$, $\varepsilon =0.15$, $F=0.1$, $\delta =0.05$, (**a**) $\zeta =0$, (**b**) $\zeta = 0.01$.

**Figure 4:**
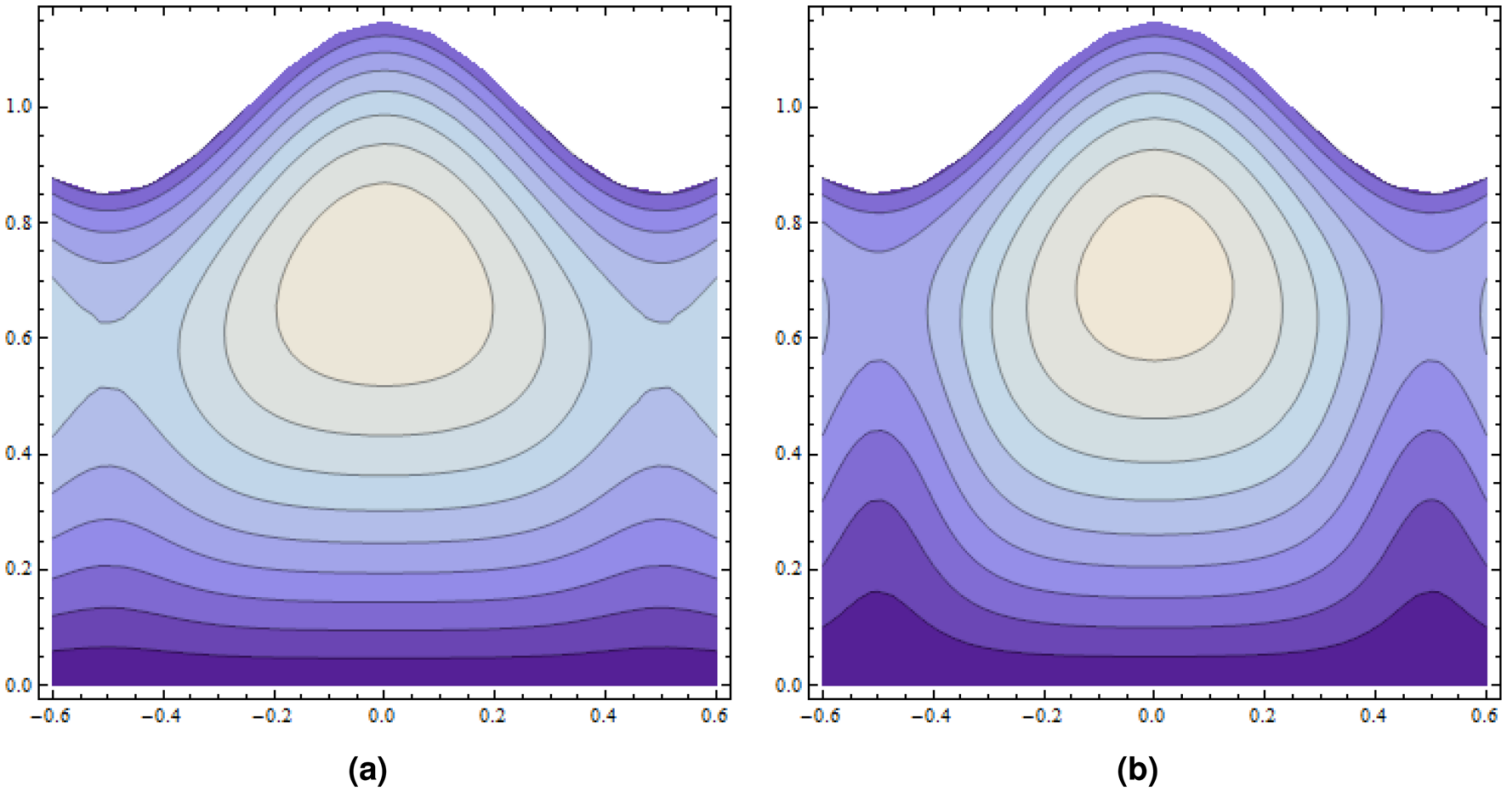
Plots of several stream lines with $\alpha =0.2$, $\varepsilon =0.15$, $F=0.1$, $\delta =0.05$, (**a**) $\zeta =0.02$, (**b**) $\zeta = 0.03$.

**Figure 5:**
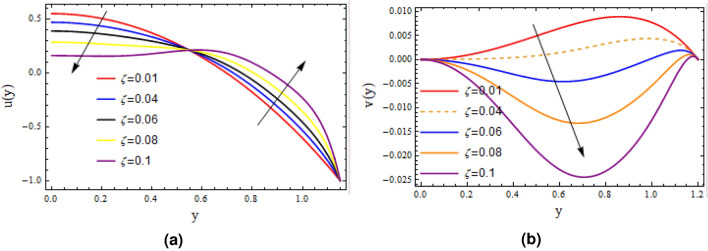
Velocity profile variations with (**a**) and (**b**) $\alpha = 0.2$, $\varepsilon = 0.15$, $\delta =0.01$, $F= 0.1$, $x= 1$.

Cilia induced flow for pseudo plastic nano fluid model is investigated. Flow is modelled by considering the long wave length theory and low Reynolds number. Solution for the proposed physical phenomenon is obtained by capitalizing the strength of perturbation technique. Analytical expressions are gathered for stream function, concentration, temperature profiles, axial velocity, and pressure gradient. Whereas, transverse velocity, wave length for pressure rise, and frictional force on the walls of the tubule are investigated with aid of numerical computations and their outcome are demonstrated graphically. Here in this section impacts of $\zeta$ relaxation time, thermophoresis parameter $N_{t}$, Prandtl number *Pr* on velocity distribution, concentration, temperature profiles, pressure gradient, wave length for pressure rise and frictional force are investigated. A comprehensive investigation in the form of numerical data has been exhibited in the Tables 1 and 2. In the first table analysis of perturb and numerical solution for axial velocity *u* is made, almost similar values of perturb and numerical solution with very small difference is recorded. In the Table 2 comparison of perturb and numerical solution for stream function is done and almost identical numerical data is obtained. Both the tables prove the authenticity of our analytical solution. Graph of axial component of velocity, for perturb and numerical solution is exhibited in Fig. 2, it is worth to mention here that we have obtained all most similar curves for both solution, which validates our analytical solution. Trapping phenomenon is exhibited in Figs. 3 and 4, it is quite evident that as relaxation time is enhanced, the number of circulating streamlines increases and some fluctuations occurs in the size of the trapped bolus. Variations on the velocity profile with enhancement in the relaxation time demonstrated in Fig. 5, it is seen that initially longitudinal component of velocity depreciates as relaxation time $\zeta$ is increased and surge is observed in the velocity because of the no slip phenomenon. Where as, mere a sharp decline is seen in the transverse component of velocity with rising values of $\zeta$. Actually relaxation time $\zeta$ is the measure of fluid inertia, because of this factor retardation in the velocity profile is recorded.

**Figure 6:**
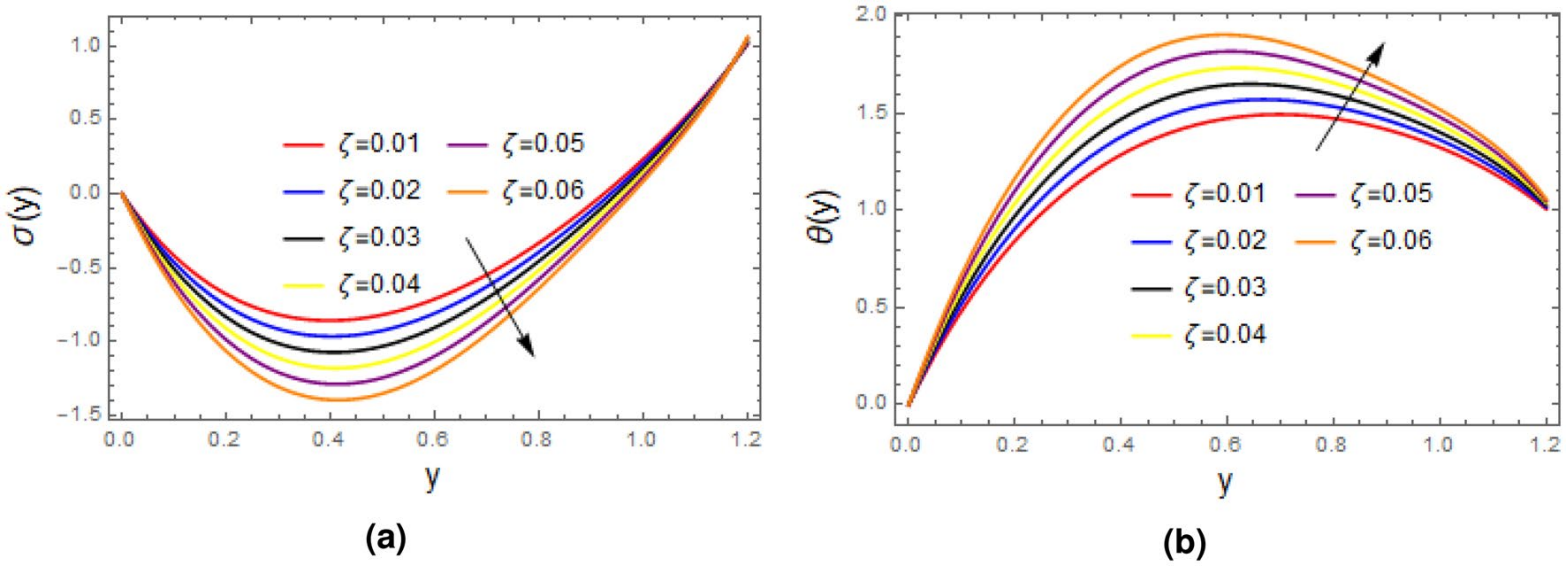
Plots of (**a**) concentration $\sigma$ and (**b**) temperature $\theta$ for variations of relaxation time, with $\alpha = 1$, $\varepsilon = 0.2$, $\delta =0.1$, $F= 0.5$, $x= 1$, $Pr= 2$, $N_{b}= 0.8$, $N_{t}= 1$, $Ec= 1$.

**Figure 7:**
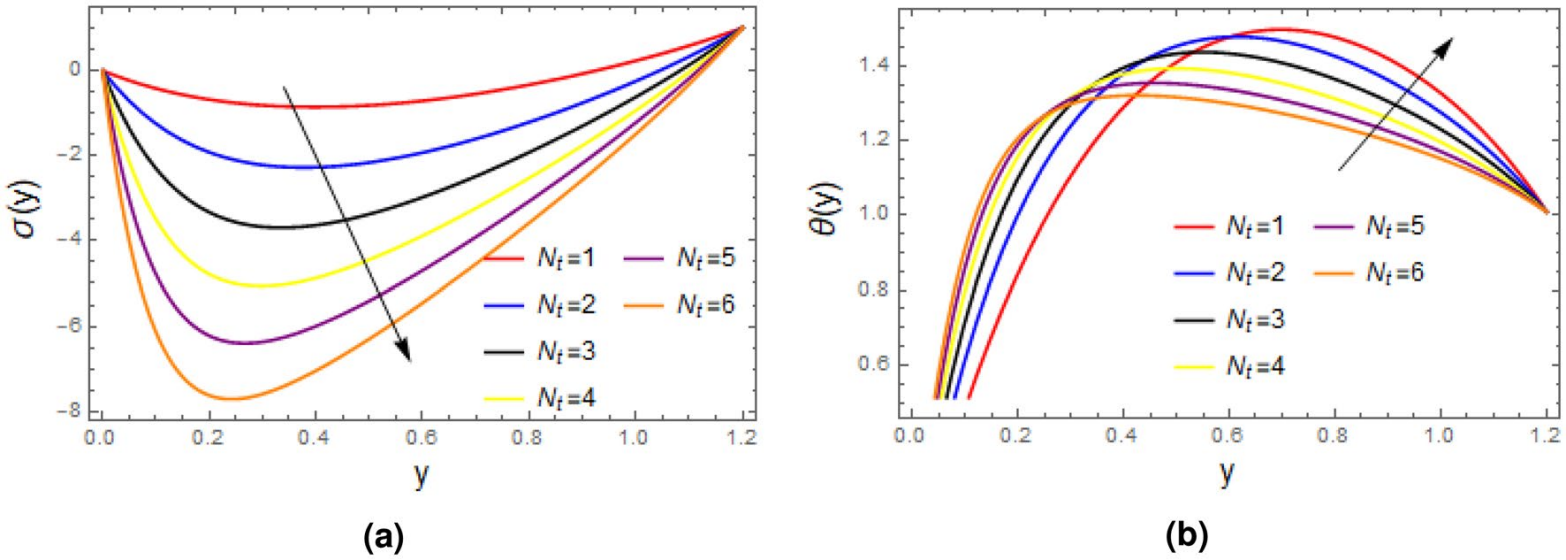
Plots of (**a**) concentration $\sigma$ and (**b**) temperature $\theta$ for different values of thermophoresis parameter with $\alpha = 1$, $\varepsilon = 0.2$, $\delta =0.1$, $F= 0.5$, $x= 1$, $Pr= 2$, $N_{b}= 0.8$, $\zeta = 0.01$, $Ec= 1$.

Figure 6 portrays the impact of relaxation time on concentration and temperature profiles, it is concluded from the plots that concentration falls as enhancement in the value of the $\zeta$ is made, while opposite behavior is observed for the temperature distribution. Figure 7 elucidate that the as thermophoresis parameter $N_{t}$ is enhanced, yields decrease in concentration and increase in temperature profile. Which happen due to fact that thermophoresis mechanism give rise to the motion of fluid elements, they collides with each other due to which energy of fluid element increases, which results increase in temperature and decline in the concentration profile.

**Figure 8:**
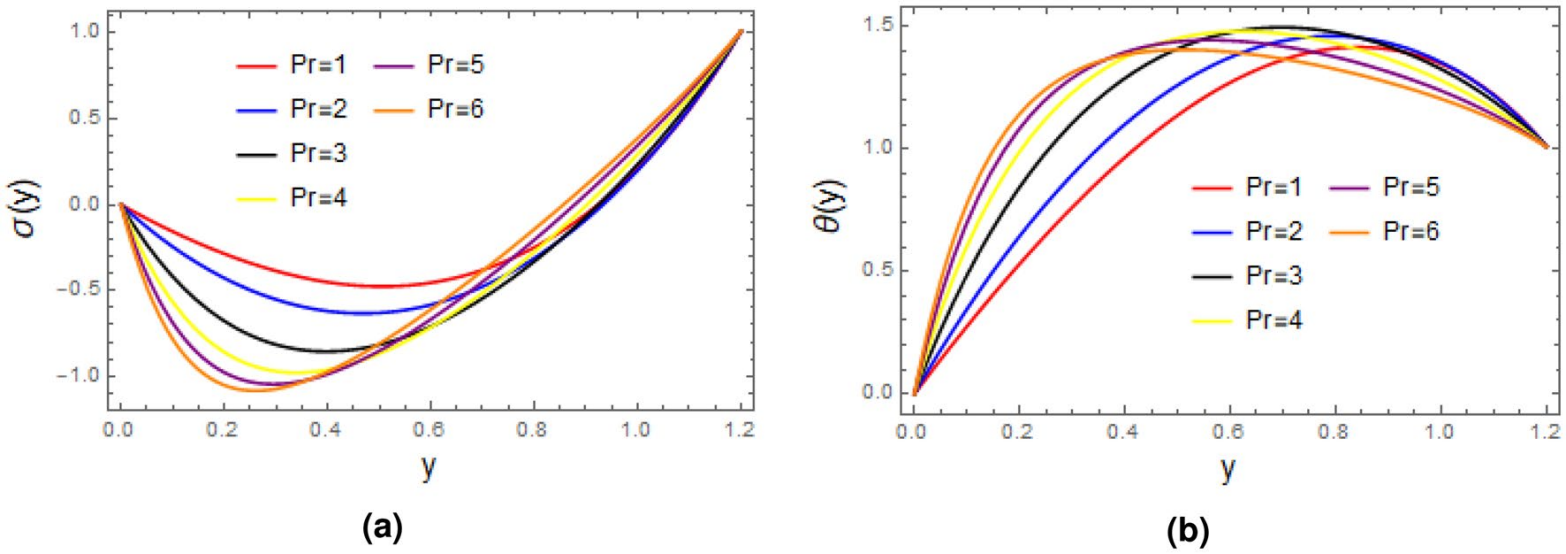
Plots of (**a**) concentration $\sigma$ and (**b**) temperature $\theta$ for Prandtle number with $\alpha = 1$, $\varepsilon = 0.2$, $\delta =0.1$, $F= 0.5$, $x= 1$, $Ec= 1$, $N_{t}= 1$, $\zeta = 0.01$, $N_{b}= 0.8$.

Effect of Prandtl number is investigated in Fig. 8, it is concluded from the first figure that concentration profile declines as Prandtle number is enhanced, which means that momentum diffusivity become weak and thermal diffusivity has dominant role. From Fig. 8b it noted that temperature profile become strong as Prandtle number is enhanced.

**Figure 9:**
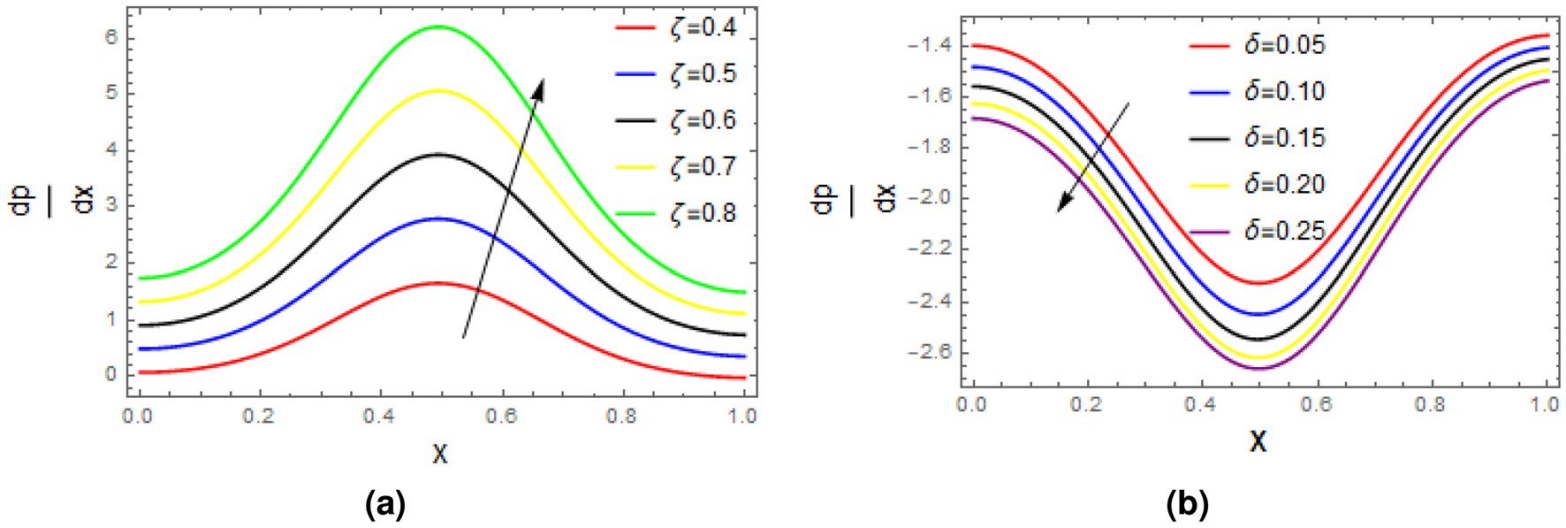
Plots of pressure gradient, impacts of $\zeta$ and $\delta$ are studied with $\alpha = 1,\varepsilon = 0.2, Q= 0.5$ (**a**) $\delta =0.05$, (**b**) $\zeta =0.05$.

**Figure 10:**
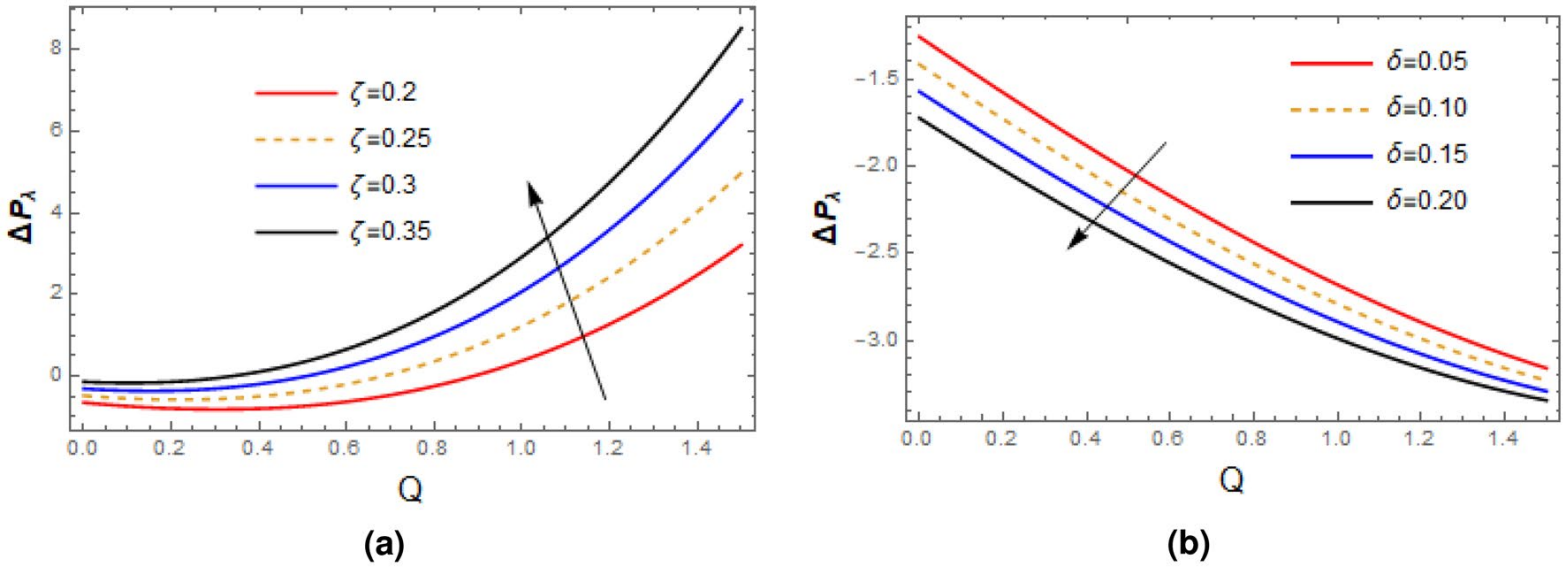
Plots of pressure rise per wave length, variations of $\zeta$ and $\delta$ are exhibited with $\alpha = 1,\varepsilon = 0.2, Q= 0.5$ (**a**) $\delta =0.05$, (**b**) $\zeta =0.02$.

**Figure 11:**
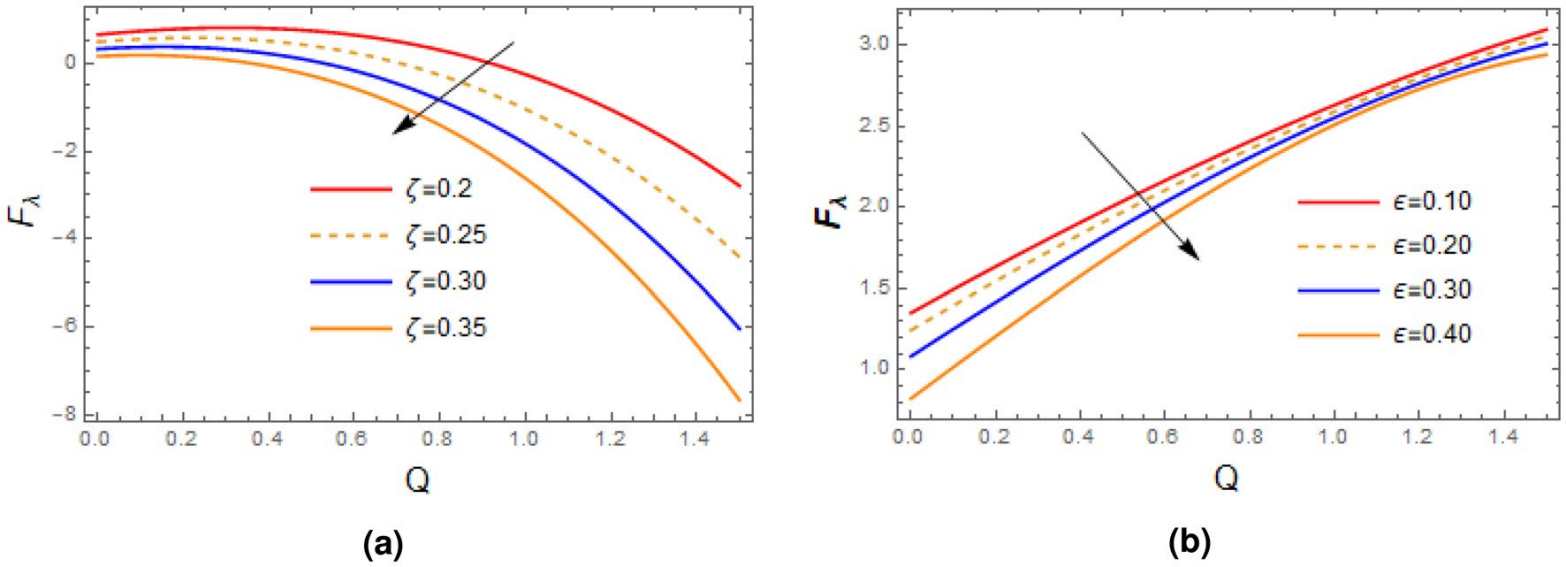
Plots of frictional force on wall of tubules, variations of $\zeta$ and $\varepsilon$ are exhibited with $\alpha = 1,\delta = 0.2, Q= 0.5$ (**a**) $\varepsilon =0.2$, (**b**) $\zeta =0.02$.

Plots of pressure gradient as function of relaxation time $\zeta$ and wave number $\delta$ are portrayed in Fig. 9, it is seen from the first figure that as the measure of fluid is raised, the pressure gradient profile enhances, on other when the wave number $\delta$ is extended, then the reverse behavior is seen. Figure 10 demonstrates the impact of $\zeta$ and $\delta$ on pressure rise based on wave length $\bigtriangleup P_{\lambda }$, remarkable rise is seen in the value of $\bigtriangleup P_{\lambda }$ with the increase in relaxation time while opposite trend is recorded for enhancing the wave number $\delta$. Impact of $\zeta$ and wall contraction/length $\varepsilon$ on friction force $F_{\lambda }$ over the wall is exhibited in Fig. 11, it is observed that friction force $F_{\lambda }$ significantly declines with the enhancement in measure of fluid inertia, and quit opposite behavior is noted for increasing wall contraction $F_{\lambda }$.

**Table 1:** Shows analysis of Perturb solution with numerical solution with $\zeta =0.01,\alpha = 0.2$, $\varepsilon = 0.15$, $\delta =0.01$, $F= 0.1$, $x= 1$.

*y*	Perturb solution for *u*	Numerical solution for *u*	Difference
0.	0.564385	0.564638	$-\,2.53724\times 10^{\text {-4}}$
0.1	0.552597	0.552843	$-\,2.4605\times 10^{\text {-4}}$
0.2	0.517232	0.517455	$-\,2.23174\times 10^{\text {-4}}$
0.3	0.458275	0.458461	$-\,1.8578\times 10^{\text {-4}}$
0.4	0.375705	0.375841	$-\,1.35904\times 10^{\text {-4}}$
0.5	0.269491	0.269569	$-\,7.76974\times 10^{\text {-5}}$
0.6	0.13959	0.139607	$-\,1.75488\times 10^{\text {-5}}$
0.7	− 0.0140506	− 0.0140875	$3.68592\times 10^{\text {-5}}$
0.8	− 0.191489	− 0.191567	$7.83305\times 10^{\text {-5}}$
0.9	− 0.392793	− 0.392894	$1.01432\times 10^{\text {-4}}$
1.	− 0.618037	− 0.618137	$1.00026\times 10^{\text {-4}}$
1.1	− 0.867319	− 0.867374	$5.51864\times 10^{\text {-5}}$

**Table 2:** Shows analysis of Perturb solution with numerical solution for stream function with $\alpha = 0.2, \varepsilon = 0.15, \delta =0.01, F= 0.1, x= 1$.

*y*	Perturb solution	Numerical solution	Difference
0.	0.	0.	0.
0.1	0.0560456	0.0560623	$-\,1.67013\times 10^{\text {-5}}$
0.2	0.109734	0.109766	$-\,3.20222\times 10^{\text {-5}}$
0.3	0.158706	0.15875	$-\,4.45712\times 10^{\text {-5}}$
0.4	0.200601	0.200655	$-\,5.31095\times 10^{\text {-5}}$
0.5	0.233058	0.233115	$-\,5.67039\times 10^{\text {-5}}$
0.6	0.25371	0.253765	$-\,5.49514\times 10^{\text {-5}}$
0.7	0.260185	0.260233	$-\,4.81658\times 10^{\text {-5}}$
0.8	0.250107	0.250144	$-\,3.74213\times 10^{\text {-5}}$
0.9	0.221092	0.221116	$-\,2.44263\times 10^{\text {-5}}$
1.	0.17075	0.170762	$-\,1.14196\times 10^{\text {-5}}$
1.1	0.0966832	0.0966849	$-\,1.73212\times 10^{\text {-6}}$

## Conclusion

In this investigation an effort is made to explore the Cilia induced flow for pseudo plastic nano fluid model which is applicable to ductus efferent of human male reproductive tract. For physiological problem, flow is modeled by employing low Reynolds number and long wave length approximation. A novel solution for the proposed physical phenomenon is obtained by capitalizing the strength of perturbation technique. Analytical expressions are gathered for stream function, concentration, temperature profiles, axial velocity, and pressure gradient. Whereas, transverse velocity, pressure rise per wave length, and frictional force on the wall of the tubule are investigated with aid of numerical computations. Key finding of the current investigation may be elaborated as:

- Circulating stream lines are remarkably increased with the enhancement in the value of fluid inertia $\zeta$.
- Velocity profile deteriorates with increasing relaxation time.
- It is studied that as value of relaxation is enhanced, the concentration profile declines and temperature profile become strong.
- Concentration profile deteriorates with thermophoresis parameter $N_{t}$ and Brownian motion parameter $N_{b}$, whereas temperature profile significantly enhances.
- Pressure rise per wave length $\bigtriangleup P_{\lambda }$ enhances appreciatively with relaxation time and decline with wave number $\delta$.
- Frictional force on the wall of the channel decreases with increasing relaxation time and contraction/length $\varepsilon$.

## Supplementary Information

Supplementary Information.
