# Supplementary material for: A novel mathematical modeling with solution for movement of fluid through ciliary caused metachronal waves in a channel
Source: Sci Rep. 2021 Oct 18;11:20601. doi: 10.1038/s41598-021-00039-6 (PMC8523557; doi:10.1038/s41598-021-00039-6)
Supplement: Supplementary file 1 — Supplementary Information. [file 41598_2021_39_MOESM1_ESM.pdf]

Appendix

$$
\begin{aligned}
\gamma_1 &= 0,\\
\gamma_2 &= -\frac{-N_b-N_t}{hN_b},\\
\gamma_3 &= -\frac{-1+\frac{3Ec\left(6+3(-N_b-N_t)\Pr+(-N_b-N_t)^2\Pr^2\right)(F-2hu_h)^2}{4h^2(-N_b-N_t)^3\Pr^3}}{-\frac{h}{(-N_b-N_t)\Pr}+\frac{e^{(-N_b-N_t)\Pr}h}{(-N_b-N_t)\Pr}}\\
\gamma_4 &= -\frac{1}{4\left(-1+e^{-(-N_b-N_t)\Pr}\right)h^2(-N_b-N_t)^3\Pr^3}e^{-(-N_b-N_t)\Pr}\left(18EcF^2+9EcF^2(-N_b-N_t)\Pr\right.\\
&\quad +3EcF^2(-N_b-N_t)^2\overset{2}{\Pr}-4h^2(-N_b-N_t)^3\overset{3}{\Pr}-72EcFhu_h-36EcFh(-N_b-N_t)\Pr u_h\\
&\quad \left.-12EcFh(-N_b-N_t)^2\overset{2}{\Pr}u_h+72Ech^2{u_h}^2+36Ech^2(-N_b-N_t)\Pr{u_h}^2+12Ech^2(-N_b-N_t)^2\overset{2}{\Pr}{u_h}^2\right)
\end{aligned}
$$

$$
\begin{aligned}
\delta_1 &= 0\\
\delta_2 &= -\frac{-N_b-N_t}{hN_b},\\
\delta_3 &= -\frac{1}{175{\delta_2}^6h^{17}{N_b}^6\Pr^6}\left(1+\frac{1}{-1+e^{\delta_2 hN_b\Pr}}\right)\left(-175{\delta_2}^7h^{17}{N_b}^7\overset{7}{\Pr}-1458Ec(63000+\right.\\
&\quad \delta_2 hN_b\Pr(-31500+\delta_2 hN_b\Pr(7980+\delta_2 hN_b\Pr(-1365+2\delta_2 hN_b\Pr(84+\\
&\quad \left.\delta_2 hN_b\Pr(-7+\delta_2 hN_b\Pr))))))(F-hu_h)^6\right)\\
\delta_4 &= \frac{e^{\delta_2 hN_b\Pr}}{175{\delta_2}^7\left(-1+e^{\delta_2 hN_b\Pr}\right)h^{17}{N_b}^7\Pr^7}\left(175{\delta_2}^7h^{17}{N_b}^7\overset{7}{\Pr}+1458Ec(63000+\delta_2 hN_b\Pr(-31500\right.\\
&\quad \left.+\delta_2 hN_b\Pr(7980+\delta_2 hN_b\Pr(-1365+2\delta_2 hN_b\Pr(84+\delta_2 hN_b\Pr(-7+\delta_2 hN_b\Pr))))))(F-hu_h)^6\right)
\end{aligned}
$$
